# Heme Binding to HupZ with a C-Terminal Tag from Group A Streptococcus

**DOI:** 10.3390/molecules26030549

**Published:** 2021-01-21

**Authors:** Ephrahime S. Traore, Jiasong Li, Tapiwa Chiura, Jiafeng Geng, Ankita J. Sachla, Francis Yoshimoto, Zehava Eichenbaum, Ian Davis, Piotr J. Mak, Aimin Liu

**Affiliations:** 1 Department of Chemistry, The University of Texas at San Antonio, San Antonio, TX 78249, USA; ephrahime.traore@utsa.edu (E.S.T.); jiasong.li@utsa.edu (J.L.); francis.yoshimoto@utsa.edu (F.Y.); christopher.davis2@utsa.edu (I.D.); 2 Department of Chemistry, Saint Louis University, St. Louis, MO 63103, USA; tapiwa.chiura@slu.edu; 3 Department of Chemistry, Georgia State University, Atlanta, GA 30302, USA; jiafeng.geng@emory.edu; 4 Department of Biology, Georgia State University, Atlanta, GA 30302, USA; ajs588@cornell.edu (A.J.S.); zeichen@gsu.edu (Z.E.)

**Keywords:** GAS, iron acquisition, heme stacking, Streptococcus, EPR, resonance Raman spectroscopy, metal-binding proteins, multimeric heme complexation, protein quaternary structure

## Abstract

HupZ is an expected heme degrading enzyme in the heme acquisition and utilization pathway in Group A Streptococcus. The isolated HupZ protein containing a C-terminal V5-His_6_ tag exhibits a weak heme degradation activity. Here, we revisited and characterized the HupZ-V5-His_6_ protein via biochemical, mutagenesis, protein quaternary structure, UV–vis, EPR, and resonance Raman spectroscopies. The results show that the ferric heme-protein complex did not display an expected ferric EPR signal and that heme binding to HupZ triggered the formation of higher oligomeric states. We found that heme binding to HupZ was an O_2_-dependent process. The single histidine residue in the HupZ sequence, His111, did not bind to the ferric heme, nor was it involved with the weak heme-degradation activity. Our results do not favor the heme oxygenase assignment because of the slow binding of heme and the newly discovered association of the weak heme degradation activity with the His_6_-tag. Altogether, the data suggest that the protein binds heme by its His_6_-tag, resulting in a heme-induced higher-order oligomeric structure and heme stacking. This work emphasizes the importance of considering exogenous tags when interpreting experimental observations during the study of heme utilization proteins.

## 1. Introduction

*Streptococcus pyogenes* (Group A Streptococcus or GAS) is an extracellular, Gram-positive, β-hemolytic bacterial pathogen that is the causative agent of various diseases, including pharyngitis, impetigo, rheumatic fever, acute glomerulonephritis, necrotizing fasciitis, and streptococcal toxic shock syndrome [1]. Over the past 40 years, a resurgence of invasive streptococcal infections and the emergence of new virulent strains have been occurring. A recent report conducted by the Center for Disease Control and Prevention of the United States named *S. pyogenes* a concerning threat due to a notable rise in erythromycin resistance [2]. Pathogenic bacteria that require iron to survive in their host typically use dedicated pathways to obtain it, such as the iron surface determinant (Isd) pathway in Gram-positive *Staphylococcus aureus* [3]. The heme uptake proteins IsdB and IsdH in *S. aureus* have multiple near transporter (NEAT) domains. The NEAT domain is comprised of 125 amino acids with a conserved tyrosine residue to ligate the heme iron [4,5]. IsdB and IsdH sequester heme from hemoglobin and transfer it between themselves as well as to other NEAT domain-containing proteins in the cell wall, such as IsdA and IsdC [5,6]. These NEAT domain-containing proteins eventually deliver the heme to the IsdDEF ATP binding cassette (ABC) transporter, which shuttles the heme into the cytoplasm [6]. The surface NEAT receptor Shr, captures heme from hemoglobin and delivers to a second NEAT protein Shp [7,8], which in turns shuttles it to the membrane SiaABC transporter [7,9,10]. Once in the cell, a heme oxygenase (HO) from the corresponding pathogen typically oxidizes the heme porphyrin ring to procure the iron ion.

**Scheme 1:**
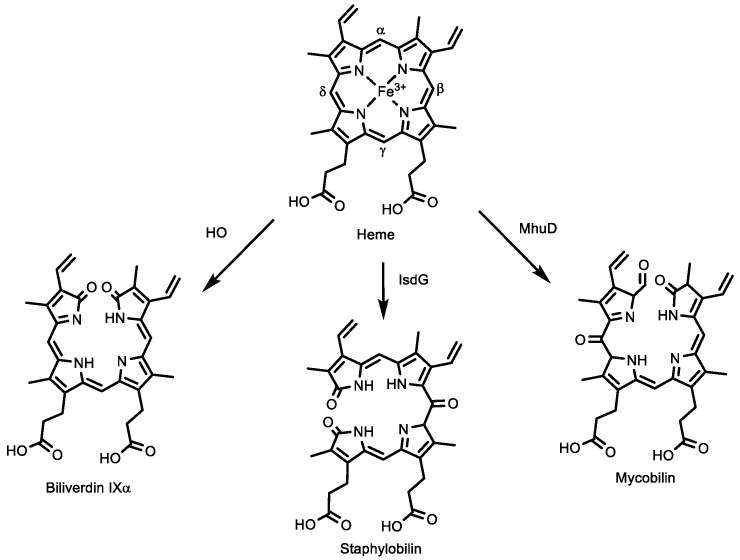
Canonical and non-canonical heme-degrading enzymes and the respective major porphyrin derivatives.

A hallmark of HOs is that these proteins employ their substrate as an enzymatic cofactor for oxygen activation and subsequent heme-oxidation reactions. The first few HOs discovered, human HO (hHO-1) [11], HmuO [12], PigA [13], and HemO [14] are described to have a “canonical heme oxidation” mechanism which utilizes 3 oxygen molecules and 7 electrons to catalyze the degradation of heme producing α-biliverdin, CO, and Fe(II) ion (Scheme 1). Another family of HOs was recently discovered and classified as noncanonical or IsdG-like HOs. This family includes IsdG, IsdI, and MhuD [15,16]. They are termed as noncanonical due to their distinctive structure and products, which suggest a novel mechanism. All previously metioned HOs bind heme with a histidine ligand. The structures of canonical HOs are comprised of α-helices and loops, while this new class of HOs is part of the ferredoxin fold superfamily in which a β-barrel fold is formed between a dimeric interface [17]. IsdG and IsdI are able to break down heme to formyloxobilin, which is further degraded into staphylobilin, releasing Fe(II) and formaldehyde as a side product. The monoheme form of MhuD catalyzes the formation of mycobilin from heme with only the release of Fe(II) and no other carbon-containing side product.

To survive, GAS must obtain iron from its host [18]. Studies have shown that GAS encodes NEAT domain-containing proteins [7,8,19,20] and an ABC transporter [7,10,21]. However, no HO homologs were found, leaving its iron acquisition pathway unresolved. In 2016, a putative heme-degrading protein was described [22]. A microarray study has revealed that the iron-dependent regulator of GAS, MtsR, represses a gene titled *spy49_0662*. The attempts to express a non-tagged gene product led to unstable or inclusion body protein. Hence, *spy49_0662* was expressed with a 1.4 kDa *C*-terminal V5 epitope tag used for Western blotting followed by a His_6_-tag for immobilized metal ion affinity purification, and the resulting protein was coined as HupZ (heme utilization protein Z) [23]. The tagged HupZ is a small-sized protein previously shown to bind heme, and it exhibits a trace heme degrading activity in the presence of NADPH and cytochrome P450 reductase (CPR). This protein shares some protein sequence similarity to the members of the IsdG family. However, CO is found to be released rather than the expected formaldehyde for a heme oxygenase from the IsdG family [23].

In this work, we revisited the putative HO activity of HupZ with the epitope and His_6_ tag at the *C*-terminus that was used in the previous study. We first obtained the EPR spectrum to understand the electronic structure of the heme iron. Interestingly, the ferric heme-bound HupZ complex yielded an unexpected EPR-silent spectrum. We then conducted a comprehensive biochemical and spectroscopic study of heme binding to HupZ to understand the chemical nature of the complex. The results show that the heme binds to the His_6_-tag in an antiferromagnetic manner between two subunits in a higher-order oligomeric structure. These findings reveal that the previously found weak heme-degradation activity is not due to a natural function of HupZ.

## 2. Results

### 2.1. The C-Terminally Tagged HupZ Presents an Unusual Heme-Protein Interaction Mode

**Figure 1:**
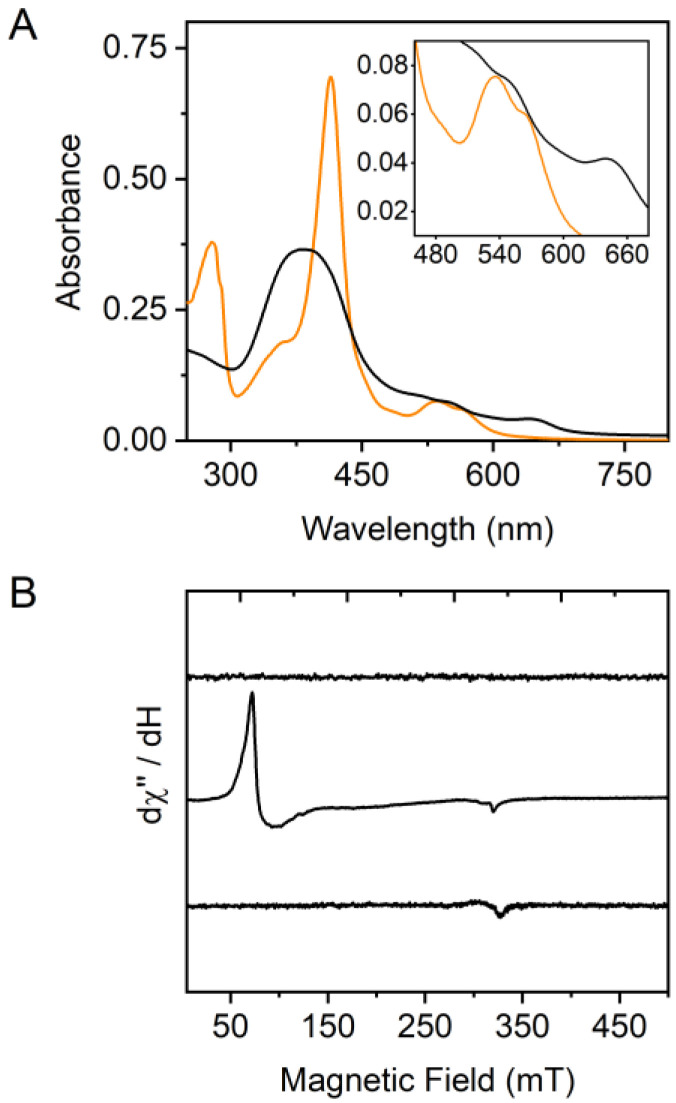
UV–vis and EPR spectra of HupZ in complex with ferric heme. (**A**) UV–vis spectra of 6 μM HupZ-heme (orange trace) compared to 6 μM free heme (black). (**B**) EPR spectra of 200 μM HupZ (top trace), 250 μM free ferric heme (middle trace), and 200 μM HupZ-heme complex (bottom trace).

The UV–vis spectrum of HupZ in complex with ferric heme is shown in Figure 1A. The complex exhibited a sharp Soret band at 414 nm, with a β band at 536 nm and an α band at 564 nm. In contrast, free ferric heme in the same buffer showed a broad Soret band centered at 385 nm with three visible bands in the Q band region. The differences in the Soret and Q band region suggested that the anticipated binary complex contains a well-behaved protein-bound heme.

The nature of heme binding to HupZ was investigated by electron paramagnetic resonance (EPR) spectroscopy. The HupZ protein alone, as expected, was EPR silent (Figure 1B). In comparison, freshly prepared hemin showed an expected axial high-spin signal with *g*_⊥_ = 5.72, *g*_‖_ = 1.99, and a ground spin state of *S* = 5/2, which is consistent with ferric hemin dissolved in *N*,*N*-dimethylformamide as described by Peisach et al. [24]. The minor resonance at approximately *g* = 4.30 resulted from adventitious iron. However, upon mixing HupZ with 1.2 eq of hemin followed by desalting to remove unbound ligand, the resulting sample was surprisingly EPR-silent even though it gave rise to an absorption spectrum identical to the orange trace shown in Figure 1A. The lack of an EPR signal from the HupZ-heme complex could be explained by: (1) the ferric heme being in a highly anisotropic low-spin (HALS) status, which presents a broadened EPR spectrum; (2) the ferric heme being reduced to an EPR-inactive ferrous state by the protein; or (3) the heme bound in such a way that two ferric heme molecules are ferromagnetically coupled resulting in an integer spin state (*S* = 5) or antiferromagnetically coupled, netting an overall *S* = 0 state. We examined the possibility of the integer spin state by using the parallel mode EPR technique, in which the modulating magnetic field is parallel to the applied field, and thus allows for the detection of transitions between eigenstates for systems with integer spin. However, the ferric heme complex with HupZ at 250 µM was spectroscopically silent in the parallel model EPR (Figure S1), indicating that a ferromagnetically coupled heme center was unlikely to be present.

### 2.2. Probing the Oxidation State of the HupZ-Heme Complex

**Figure 2:**
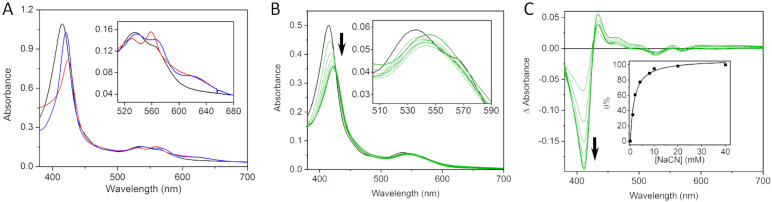
Oxidation state of heme in HupZ complex monitored by UV–vis spectroscopy. (**A**) the binary complex of 10 μM HupZ-heme (black), reduced HupZ-heme complex with 1 mM dithionite (red), and CO adduct of the reduced HupZ-heme complex (blue). (**B**) HupZ (5 μM)-heme complex (black) titrated with NaCN up to 2 mM (green). (**C**) Difference spectra of panel B with representative titration steps shown as thin green lines and the final 2 mM NaCN shown as a thick green line. The inset depicts the percent bound of CN to heme as a function of the concentration of NaCN added.

The EPR observation seemingly contradicts the UV–vis spectrum of the HupZ-heme complex. To understand the chemical nature of the heme bound in HupZ, we must first determine the oxidation state of the heme iron in the binary complex. Thus, we probed the oxidation state of the HupZ-heme complex with carbon monoxide (CO), as it is among the strongest ligands exclusively for ferrous but not ferric heme. Exposure of the HupZ-heme complex to CO did not cause any detectable spectral changes, suggesting the heme in HupZ remained in the ferric form. The addition of dithionite as a reducing agent led to expected heme reduction with loss of the Soret band intensity and red-shift from 414 to 424 nm. The α/β bands became sharper and showed a slight blue shift to 559 and 530 nm, respectively (Figure 2A, red trace). Subsequent addition of CO to the reduced HupZ-heme generated additional spectral changes corresponding to an expected ferrous-CO complex, with the Soret band increasing in intensity and blue-shifting by 3 nm (Figure 2A, blue trace). The α/β bands broadened and showed a red-shift to 567 and 537 nm, respectively. Additionally, a well-defined charge transfer band at 623 nm was observed. The HupZ-heme complex has spectral characteristics that resemble histidine-ligated heme proteins, such as Soret band at 421 nm for the Fe(II)-CO complex [25]. As an additional probe of the HupZ-heme complex oxidation state, we generated the cyano complex of the ferric heme (Figure 2B). As the binary HupZ-heme complex was titrated with NaCN, the Soret band decreased its intensity, red-shifting to 416 nm with a new Q band at 543 nm. All UV–vis characteristics of the HupZ-heme complex are shown in Table 1. The difference spectrum of the NaCN titration (Figure 2C) showed the most significant difference at 415 nm. Plotting this difference as the percentage of CN bound to heme versus the concentration of NaCN added allowed the determination of a *K*_D_ of 18.7 ± 1.07 μM for cyanide binding. At higher NaCN concentrations, above 2 mM NaCN, the Soret band increased and gradually red-shifted to 423 nm (Figure S2). In this later phase, the intensity of the Soret band increased linearly as the concentration of NaCN increased; thus this phase was not used for the calculation of *K*_D_.

### 2.3. Resonance Raman Spectroscopy Suggests a Six-Coordinate Low-Spin Heme with Histidine Axial Ligand(s)

**Figure 3:**
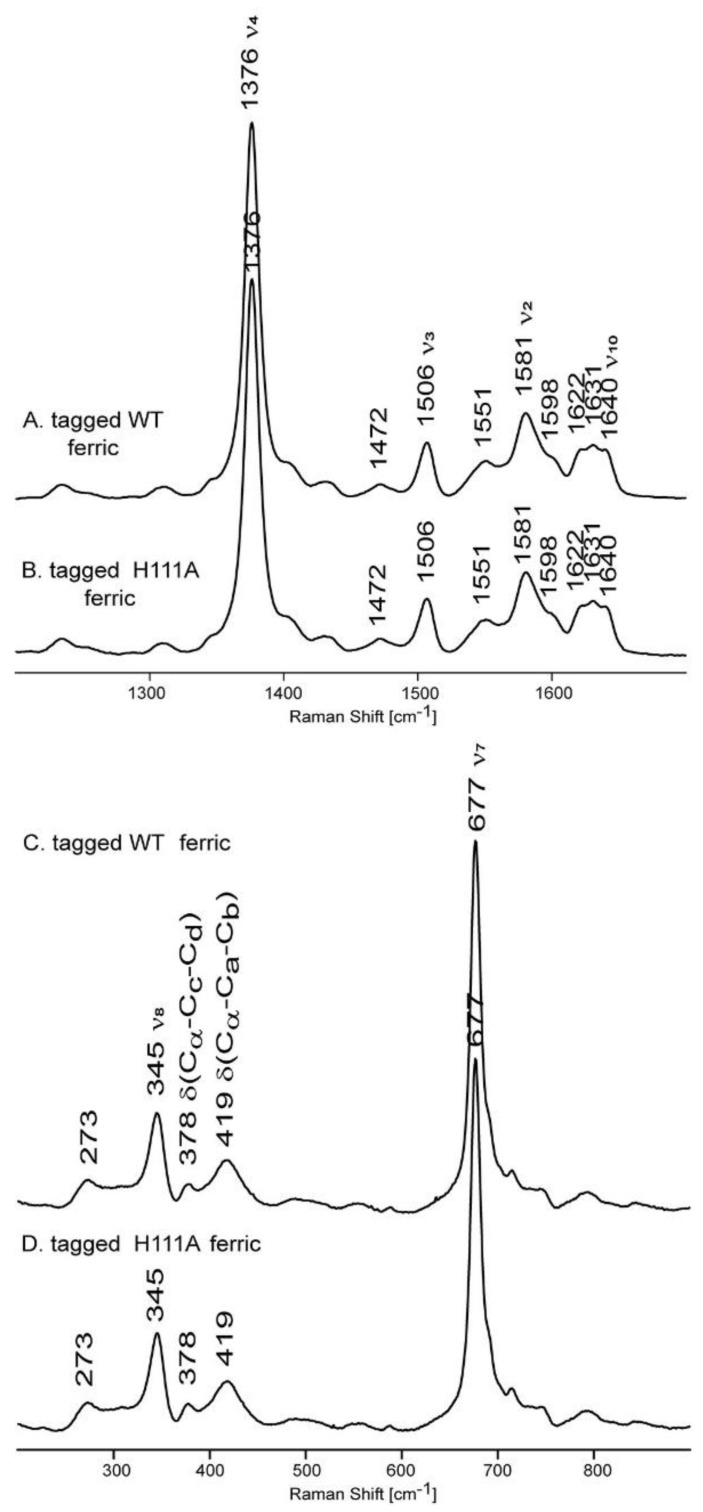
The resonance Raman (rR) spectra of HupZ and the H111A variant. (**A**) Ferric HupZ-heme complex and its (**B**) H111A mutant in the high frequency region, and the corresponding spectra in the low frequency region (**C**,**D**). All samples were measured with 406 nm excitation line at room temperature.

To further probe the heme-binding mode in the binary complex, a resonance Raman (rR) spectroscopic characterization was executed. Figure 3 shows the high- (top panel) and low-frequency (bottom panel) rR spectra of HupZ-heme complex prepared in aerobic conditions. The rR spectrum exhibits the ν_4_ oxidation state marker band at 1376 cm^−1^, a frequency typical for the ferric state. The positions of the spin state marker bands, the ν_3_ mode at 1506 cm^−1^, ν_2_ mode at 1581 cm^−1^, and ν_10_ mode at 1640 cm^−1^ indicate a six-coordinate low-spin state of the heme iron. The low-frequency rR spectrum of the HupZ-heme complex exhibits dominant ν_7_ and ν_8_ modes at 677 cm^−1^ and 345 cm^−1^. The propionate and vinyl bending modes are seen at 378 cm^−1^ and 419 cm^−1^, respectively. The spectral patterns are similar to that of the histidine-ligated globins and oxygenases, an observation consistent with the conclusion derived from analysis of the high-frequency spectral region. 

**Figure 4:**
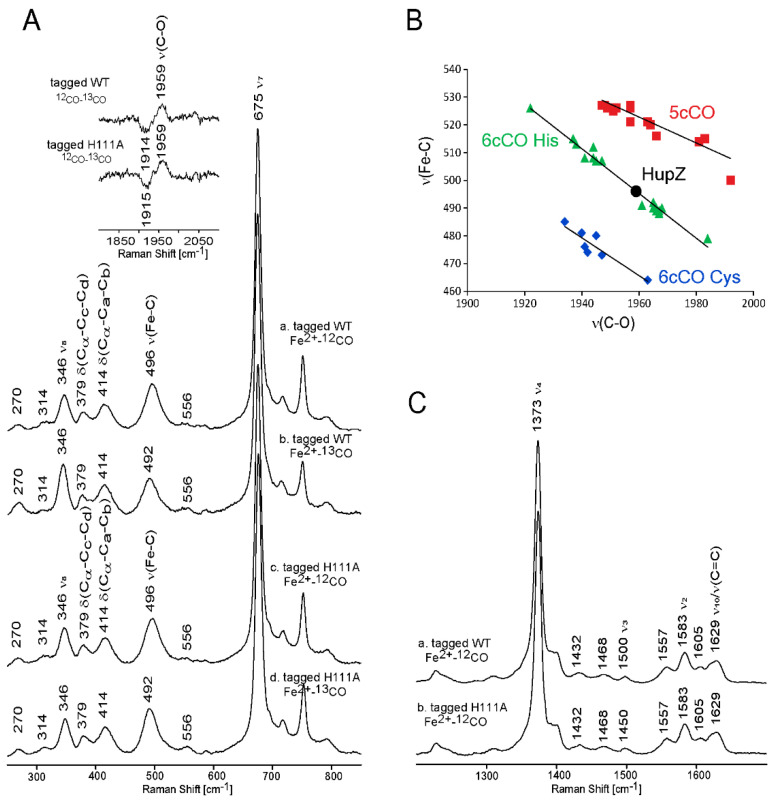
Identification of the axial ligand of heme by rR spectroscopy. (**A**) The low-frequency resonance Raman spectra of ferrous CO adducts of wild-type HupZ, (**a**) Fe^2+^-^12^CO and (**b**) Fe^2+^-^13^CO as well as H111A variant, (**c**) Fe^2+^-^12^CO and (**d**) Fe^2+^-^13^CO. The inset shows the ^12^CO-^13^CO difference traces of wild-type HupZ and H111A variant in the region where the ν(CO) modes are observed. (**B**) The ν(Fe-C)/ν(C-O) inverse correlation plot with lines characteristic for six-coordinated CO adduct of histidine ligated proteins (green triangle), five-coordinated CO adducts (red squares), and six-coordinated CO adducts of cysteine ligated proteins (blue diamonds); the data point for the binary complex of wild-type HupZ and H111A variant with bound heme is marked by black circle. (**C**) The high-frequency region of the ferrous CO adducts of (**a**) wild-type HupZ and (**b**) H111A variant. The reduction of ferric HupZ protein was achieved by anaerobic addition of freshly prepared sodium dithionate in a 3–5-fold molar excess. The formation of the ferrous CO adducts was done by addition of a few mL of natural abundance CO gas or the ^13^CO isotope analogue to the ferrous samples.

The low-frequency spectra of ferrous-CO adducts of anaerobically prepared HupZ-heme are shown in Figure 4A. The ν(Fe-C) stretching mode is seen at 496 cm^−1^, a frequency close to that observed for histidine ligated proteins. The assignment of this band is further confirmed by its isotopic shift to the lower frequency upon substitution of natural abundance CO gas with its ^13^CO isotope; e.g., the 496 cm^−1^ mode shifts to 492 cm^−1^. The 4 cm^−1^ isotopic shift upon ^12^CO/^13^CO substitution is consistent with previously published data for Mb and HO proteins. The corresponding ν(C-O) stretching mode is observed at 1955 cm^−1^ and shifts to 1914 cm^−1^ for the ^13^CO sample; the positions of the positive and negative features in the difference traces (Figure 4A) exhibit expected isotopic sensitivity. The frequencies of modes associated with the Fe-C-O fragment can be plotted on the ν(Fe-C) and ν(C-O) inverse correlation graph. As seen in Figure 4B (green triangles), the ν(Fe-C)/ν(C-O) point of HupZ-heme is located on the line characteristic for the histidine ligated proteins [26], providing convincing evidence that the heme in HupZ is coordinated by a histidine residue in the CO-bound heme complex in HupZ. The high-frequency spectrum of the ferrous-CO adducts of the HupZ protein is shown in Figure 4C, trace a; the ν_4_ mode and the ν_3_ modes are seen at 1373 cm^−1^ and 1500 cm^−1^, respectively. Collectively, the rR data coupled with the UV–vis study indicated that the heme in the binary complex is in a six-coordinate, low-spin ferric state with at least one histidine as an axial ligand.

### 2.4. The Role of Dioxygen in HupZ-Heme Interactions

**Figure 5:**
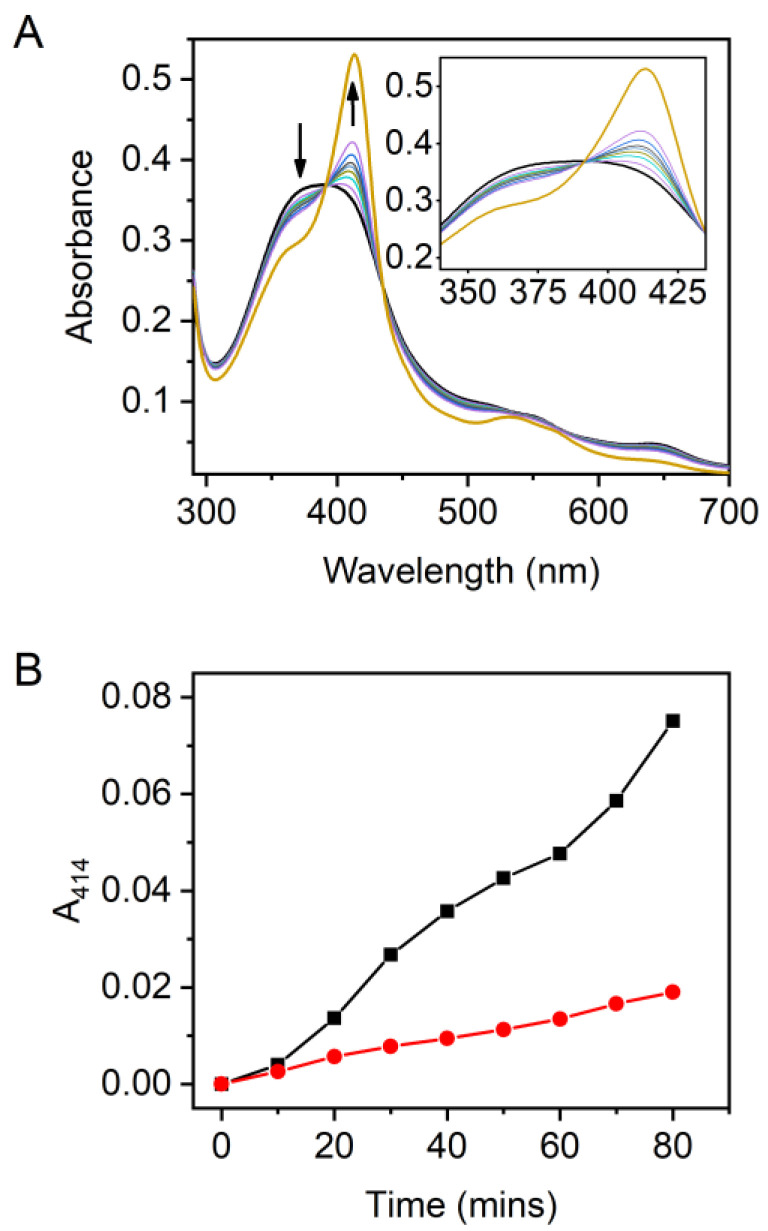
An O_2_-dependent heme binding to HupZ. (**A**) Aerobic reconstitution of 5 μM HupZ with 5 μM heme over 4 h with the 4 h spectrum drawn in orange. The arrows indicate the trend of change in the spectra (**B**) Difference spectra monitoring 414 nm absorbance plotted against time for aerobic (black squares) and anaerobic (red circles) plotted against time.

Next, the formation of the binary HupZ-heme complex was tested aerobically and anaerobically to determine if dioxygen had a role in heme binding to HupZ. Both HupZ and hemin were prepared anaerobically as described in the Materials and Methods. Aerobic reconstitution of heme over 4 h showed a noticeable increase in the Soret peak at 414 nm, indicating that heme is binding to HupZ (Figure 5A). A peak was only beginning to become visible at 414 nm in a parallel experiment under anaerobic conditions, suggesting that the lack of molecular oxygen prohibited heme from effectively binding to HupZ (Figure S3B). Monitoring the 414 nm of aerobic and anaerobic reconstitution and plotting the difference against time yielded Figure 5B. An evident rise was observed in aerobic binding, while a slow rise was noticed under anaerobic conditions. The reconstitution rate difference implies that dioxygen plays a role in facilitating heme loading and anaerobic conditions prevent the formation of the HupZ-heme complex. The slow rise of the 414 nm peak seen during anaerobic reconstitution could result from oxygen lethargically leaking into the anaerobic cuvette over the 10-h period. Under strictly anaerobic conditions, no increase of the Soret band could be detected (Figure S3C), re-affirming the necessity of dioxygen to facilitate the specific, tight binding of heme to HupZ. To understand heme binding, HupZ was titrated with hemin under aerobic conditions to determine the stoichiometric ratio of heme to protein. Our results (Figure S4) indicate that each subunit of HupZ binds heme in a 1:1 ratio. 

### 2.5. Side-Directed Mutagenesis Analysis for Identification of the Axial Ligand

Because our UV–vis and rR data suggested a histidine as an axial ligand, we turned our attention to His111 in HupZ, which is the only histidine residue aside from the *C*-terminal tag. We mutated His111 to probe its potential role in heme binding. The H111A variant was expressed and purified in the same manner as wild-type HupZ (Materials and Methods). The UV–vis spectrum of the heme-bound H111A variant is nearly identical to wild-type HupZ (Figure S5A). H111A in complex with heme was EPR silent (Figure S5B), as observed in the binary complex of wild-type HupZ, indicating mutation of His111 did not lead to an observable change of the heme-center electronic structure. Likewise, the high- and low-frequency rR spectra of H111A variant are virtually identical to that of the wild-type (Figure 3). The oxidation and spin state markers are seen at 1376, 1506, 1581, and 1640 cm^−1^ for the ν_4_, ν_3_, ν_2_, and ν_10_ modes, respectively. The analysis of the data in the low frequency also revealed that there are no significant changes between the H111A variant and wild-type HupZ in the disposition of peripheral heme groups, or the degree of out-of-plane heme macrocycle deformation, implying that the heme is coordinated by the mutated protein in a similar manner to that of wild-type HupZ. Since H111A and wild-type HupZ show nearly identical UV–vis, EPR, and rR spectra, His111 is not a heme ligand for HupZ. The only remaining histidine residues are present in the *C*-terminal tag; therefore, we conclude that the heme binding to HupZ occurs at His_6_-tag.

### 2.6. Heme-Induced Higher-Order Oligomerization in Protein Quaternary Structure

**Figure 6:**
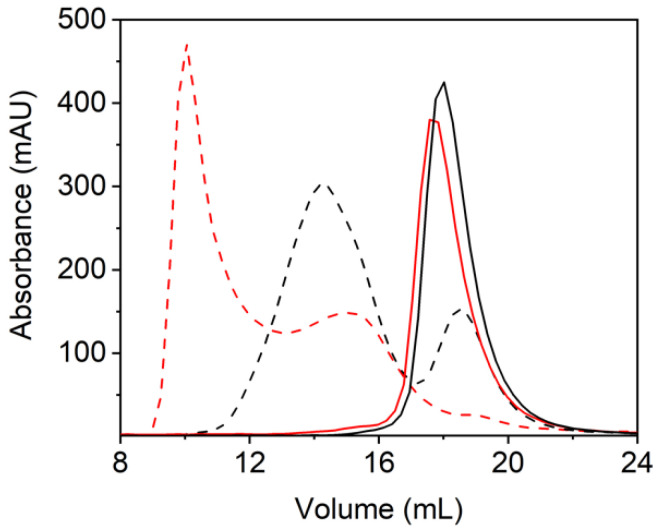
Heme-induced protein quaternary structure. SEC profile of HupZ (**solid black**), HupZ-heme binary complex (**dashed black**), H111A variant **(solid red**), and heme-bound H111A variant (**dashed red**).

The influence of heme on the oligomeric states of HupZ was investigated via size-exclusion chromatography (SEC) using an analytical Superdex-200 column (see Section 4. Materials and Methods). The separation profile of the ligand-free HupZ and the heme-bound HupZ is shown in Figure 6. The ligand-free HupZ eluted at 18.0 mL, corresponding to a molecular weight of 36 kDa when compared to the calibration curve using the known molecular standards on the same column under the same separation conditions. The molecular weight of monomeric HupZ is 18.5 kDa, and thus it eluted as a dimer. The binary complex of HupZ-heme eluted with two components: The first eluted at 14.3 mL had an estimated molecular weight of 135 kDa, which corresponds to a heptamer, and the second eluted at 18.6 mL was consistent with a dimer. In all experiments, the higher-order oligomeric states were observed only when heme was present, indicating that heme binding to HupZ induces oligomerization, resulting in higher-order structures.

The SEC study of H111A HupZ alone eluted at 17.7 mL, which is also consistent with a dimer (Figure 6). The H111A-heme complex eluted with three fractions at 10.0, 15.1, and 18.6 mL, respectively. The first fraction eluted in the void volume of the column, suggesting aggregated protein having a molecular weight above 200 kDa. The second eluent corresponds to 101 kDa, suggesting a pentamer, and the last fraction is consistent with the dimer of heme-bound H111A HupZ. A summary of each SEC peak and its corresponding oligomeric state can be found in Table S1. As observed in wild-type HupZ, heme binding to H111A induces higher-order HupZ structures. The difference in SEC between the heme complex of wild-type HupZ and the H111A variant suggests that His111 is exposed to surface or involved in subunit-subunit interactions; and thus, mutating such a polar residue facilitated the formation of high-order oligomer structures.

**Figure 7:**
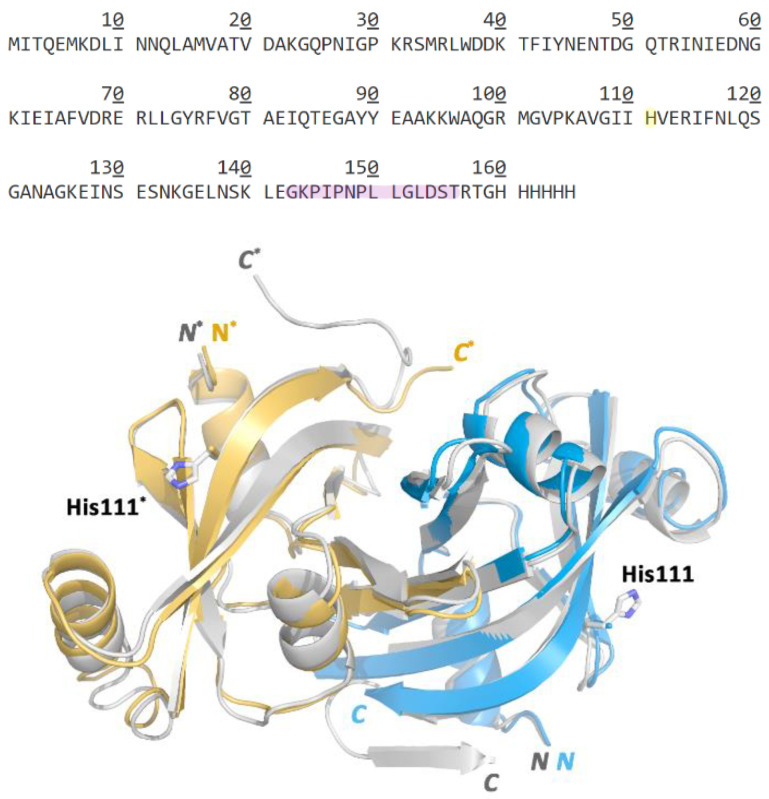
Superposition of the crystal structure of wild-type (PDB entry: 7KPZ) and H111A HupZ (7KQ2). Wild-type HupZ is shown in gray color. Chains A and B of the H111A variant are in blue and orange, respectively. His111 residues in wild-type HupZ and Ala111 residues in the H111A variant are shown as sticks with the His111 residues labeled. The *N* and *C* terminus of wt and mutant are labeled. Chain B residues and are labeled with ^*^. The top image is the amino acid sequence of HupZ with His111 highlighted in yellow and the 14-residue V5 epitope tag highlighted in purple.

Next, HupZ was crystallized in the primitive orthorhombic space group of *P*2_1_2_1_2_1_, and the structure was refined to 1.7-Å resolution (Table 2) with one dimer in an asymmetric unit (Figure 7). This structure is superimposable with the previously reported structure (PDB: 5ESC), which was crystallized in the triclinic *P*1 space group and determined at 2.0-Å resolution [23], with an r.m.s.d. value of 0.72 Å over 238 Cα carbons. In this new structure, six and seven additional *C*-terminal residues are present in Chains A and B, respectively, compared to the previous structure. The *C*-terminal region, including the His_6_-tag, still misses density for 39–40 residues. The H111A variant was crystallized in the *P*6_5_22 hexagonal trapezohedral space group, and its structure was solved and refined to 1.98-Å resolution. Comparing the structure of H111A to the wild-type structure, the two superimpose well with an r.m.s.d value of 0.99 Å over 237 Cα carbons, indicating site-directed mutagenesis did not induce an undesired global structural perturbation. His111, as shown in Figure 7, is located on the protein surface in the homodimeric structure. These structural data provide a molecular basis for comparing the biochemical and spectroscopic outcomes between wild-type and H111A HupZ.

### 2.7. The Heme-Degradation Activity of HupZ and H111A

**Figure 8:**
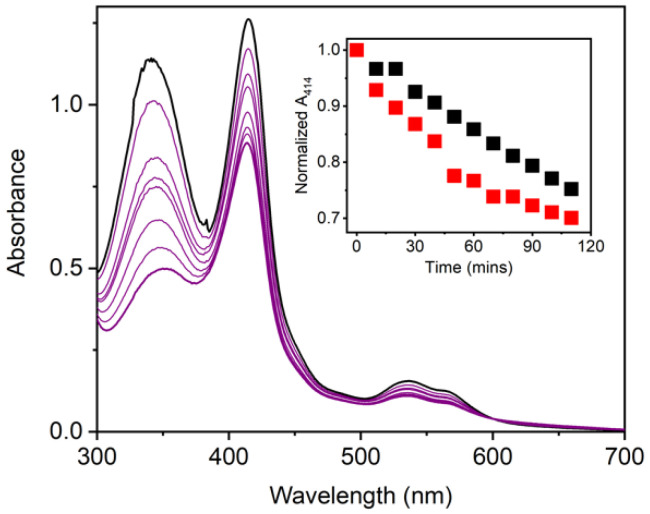
Heme degradation assay. UV–vis spectra showing heme degradation over time in the H111A variant (10 μM). The inset shows the optical absorption at 414 nm as a function of time (min) during the heme degradation reactions in wild-type HupZ (**black squares**) and the H111A variant (**red circles**).

Utilizing UV–vis spectroscopy, heme degradation was previously detected with HupZ by monitoring the decrease of the 414 nm Soret band for 5 h upon the addition of NADPH and a cytochrome P450 reductase [23]. We conducted a similar assay with wild-type and H111A HupZ for 2 h. The Soret band was observed to decrease over time for both wild-type (Figure S6) and H111A HupZ (Figure 8) on the minutes-to-hours time scale with no appreciable difference for the initial rates.

## 3. Discussion

Our experimental data collectively show that the weak heme-degradation activity found in wild-type HupZ and the H111A variant is derived from the heme bound at the *C*-terminal tag. These results draw into question whether HupZ is a heme-degradation enzyme and calls for future work on a full-length, tag-free form of HupZ, once it becomes successfully expressed.

The HupZ-heme complex did not exhibit any detectable EPR signal, which is unexpected considering the UV–vis and rR spectra. Thus, we considered several possibilities and conducted a suite of spectroscopic and structural experiments to understand how heme binds to HupZ and why the ferric heme is EPR-invisible in the protein. Three hypotheses were proposed and explored: the first is that the heme iron is being reduced by the protein and remains in the ferrous form even in the presence of air; the second presents the heme iron as a highly anisotropic low-spin (HALS) species that has a broadened EPR signal beyond detection; the third possibility proposes a diheme complex in which the two heme molecules are antiferromagnetically coupled to each other, resulting in an *S* = 0 ground state which is EPR-silent. The proposal that the ferric heme becomes reduced upon binding HupZ is excluded based on our finding that the HupZ-heme complex readily binds cyanide, but binding of CO requires prior reduction by dithionite.

A second explanation for the EPR-silent observation is that the ferric heme in the HupZ -heme complex is bound with two strong ligands in a highly anisotropic low-spin (HALS, *S* = 1/2) state. If the heme happens to have the two aromatic axial ligands with planes closer to either 0° or 90° in their relative orientation [29], it may form a HALS species [24,30,31,32]. The low-spin HALS EPR signal is typically broadened, so much that it appears to be nearly EPR invisible. However, a HALS species at a higher concentration, such as that in this work, is unlikely to present a completely silent EPR spectrum. Typically, a heme iron in HALS status would show some sort of low-spin signal as g value of 2.8–3.0 [29], but none is detected. Another piece of evidence that is inconsistent is the slow binding and the requirement of dioxygen for heme binding to HupZ. HALS requires two strong axial ligands; thus, the heme-binding process to the intended binding site is usually rapid. The final HALS complex is typically less reactive to small external molecules. In contrast, we show that heme binding to the tagged HupZ protein is a slow process strictly dependent on the presence of dioxygen. If the heme iron in HupZ is indeed in a HALS statue, then there would be no available coordination site that allows molecular oxygen to bind. 

**Scheme 2:**
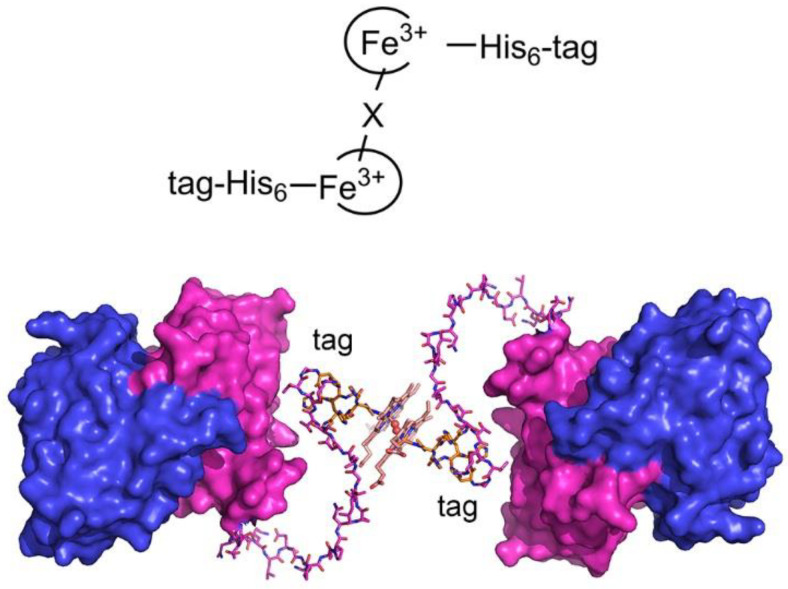
Proposed model of HupZ-heme complex with X representing a bridging ligand, and an example of which is illustrated in a cartoon constructed from the homodimeric form of the protein.

Hence, a spin-coupled heme stacking is proposed (Scheme 2), as previously found in a heme utilization pathway protein in another virulent pathogen [33] and other heme-binding proteins such as cytochrome *c* from *Geobacter sulfurreducens* [34] and cytochrome *c* nitrite reductase from *Wolinella succinogenes* [35]. We also anticipate a bridging ligand (X) to form the six-coordinate low-spin heme, although the identity of which remains unknown. Ferric ion cannot bind O_2_ under normal conditions. However, the formation of a ferric heme-O_2_ complex does occur under unusual conditions, such as a cooled ion trap [36]. It is tempting to propose a µ-oxo (or hydroxo) bridge, formed through O_2_ oxidation of a protein-reduced heme. However, no reducing agent is present. HupZ does not contain a cysteine residue with an oxidizable free thiol to reacts with oxygen; therefore, an O_2_-induced structural change due to protein oxidation is not considered as a possibility. Our observed O_2_ dependence during complex formation is directly related to the heme complexation. Since the heme-binding process is a slow event, i.e., approximately 4 h for forming the binary complex, it is too slow for a regular reduction/oxidation event. Another less common scenario plausible is the partial reduction of the ferric ion by its ligand, such as the porphyrin, causing O_2_ binding to shift the equilibrium and subsequent formation of a di-iron complex in an O_2_-dependent oxidation reaction as shown in a model study [37]. Although the current data do not identify the specific bridging ligand, it is derived in an O_2_-dependent process [38]. In principle, the bridging ligand could be identified by conducting isotope-labeling rR experiments with H_2_^18^O, and ^18^O_2_ under anaerobic conditions. However, the very slow formation of the complex may render ^18^O-scrambling to a greater extent so that it is difficult to discern. Since the overall conclusion of the multimeric heme complexation is not related to HupZ, but to the added tag, further identification of the bridging ligand was not pursued.

The protein-derived ligand assignment to the bound heme in HupZ is a result of the combination of spectroscopic and site-directed mutagenesis studies. The Soret band position of the ferric (414 nm), ferrous (424 nm), and ferrous-CO complex (421 nm) in HupZ suggests a histidine ligated heme. Importantly, the resonance Raman study provided vital information regarding the chemical nature of the heme in HupZ and in agreement with the UV–vis data. The rR spectral patterns of ferric and ferrous-CO complexes closely resemble histidine-ligated globins and heme oxygenases. The ferric complex rR data indicate that the wild-type HupZ and the H111A variant are virtually identical and are in six-coordinate low-spin states. The rR studies of ferrous-CO complexes of WT and H111A HupZ, including their isotopically substituted analogs, places the ν(Fe-C)/ν(C-O) points on the inverse correlation line characteristic for histidine ligated proteins (Figure 4); e.g., other proximal ligand candidates, such as Tyr residue or OH^-^/H_2_O, would result in a different location of the ν(Fe-C)/ν(C-O) point on the inverse correlation plots. There have been several other instances where non-enzymatic degradation of heme has led to the mis-annotation of heme-binding proteins [39,40,41]. Thus, it was crucial for us to interrogate the current form of HupZ and elucidate the nature of its heme degradation. We noted that the H111A variant had similar activity comparable to that of wild-type HupZ (Figure 8), suggesting that His111 does not contribute to the observed activity. The combination of these spectroscopic results and the functional and mutagenesis studies provides convincing evidence that the six-coordinate heme in HupZ has at least one histidine ligand and that this histidine ligand is most likely provided by the His-tag, not His111. Additional studies on the tag-free protein construct are planned to further reinforce these conclusions.

HupZ displayed a propensity of heme-induced higher-order oligomerization, causing a drastic change of the protein quaternary structure. Similar heme-induced polymerization has also been reported in SpyB, another protein in GAS recently discovered to be involved with the composition of the cell wall by Edgar et al. [42]. It has been shown when SpyB was mixed with hemin, the protein became unstable. To circumvent the unstable heme-bound SpyB, maltose binding protein (MBP) was expressed with SpyB. When MBP-SpyB was mixed with heme at a 1:4 ratio of protein:heme, the SEC profile showed two distinct peaks: the first corresponding to a heme-induced dimer and the second peak corresponding to the monomer of apo-MBP-SpyB. Likewise, heme-induced polymerization has also been described in DGCR8, which contains a heme-binding region that enhances specificity and efficiency of the formation of a microprocessor for regulating miRNAs once heme is bound [43,44]. It was determined that heme was required before the polymerization of one Drosha subunit and two DGCR8 subunits to form a microprocessor. Here, we found that HupZ had the ability to at least dimerize in the presence of heme and dioxygen, and if the heme bound to one monomer were close enough to interact with the heme of another monomer, they could stack on one another, as previously found by X-ray crystallographic study of a NEAT domain of IsdH from *Staphylococcus aureus* [33]. Due to the involvement of molecular oxygen, however, the heme stacking and protein oligomerization in HupZ is likely a concerted process.

Together, the data presented in this work favor a model by which the EPR-invisible spectrum derives from an O_2_-bridged, antiferromagnetically spin-coupled, di-heme involving two subunits of HupZ (Scheme 2). This model is consistent with the dioxygen requirement for heme loading to HupZ, the heme-induced higher-order oligomeric structures, and the EPR-silent nature of the bound heme in the ferric state. The only genetically coded histidine, i.e., His111, was shown to be irrelevant to heme binding based on our mutagenesis study. Because the UV–vis and rR data are consistent with a histidine ligated heme center, it is reasonable to conclude that the heme in the binary complex binds to the *C*-terminal His_6_-tag. These results also emphasize the importance of considering exogenous protein tag(s) when interpreting experimental observations, as previously noted in the studying of a heme-utilization protein in *Mycobacterium tuberculosis* [45]. In the heme-degrading enzyme MhuD from mycobacteria, the *C*-terminal His_6_-tag interferes with heme-binding even though no interactions between heme and the tag were observed in the X-ray crystal structures of the enzyme in complex with heme [39,40,41,46].

## 4. Materials and Methods

### 4.1. Cloning, Expression, Purification of HupZ and H111A Variant

The pZZ2 plasmid containing HupZ used for protein expression has been described previously [23]. The *Escherichia coli* BL21 (DE3) cells (Merck) containing the expression plasmid for HupZ were cultured in Luria-Bertani medium with ampicillin (100 μg/mL) at 37 °C. Upon reaching an OD_600_ of 0.8, isopropyl β-D-1-thiogalactopyranoside was added to a final concentration of 500 μM to induce protein expression, the temperature was lowered to 25 °C, and the culture was incubated for an additional 18 h. Cells were harvested by centrifugation at 6000× *g* and resuspended in 50 mM Tris-HCl, 200 mM NaCl buffered to pH 8.0. Protein was released by cell disruption (LS-20, Microfluidics), and the cell debris was removed via 45 min of centrifugation at 34,000× *g*. The debris-free supernatant was applied to a Ni-charged affinity chromatography column (GE Healthcare) and washed with 20 mM followed by 50 mM imidazole. The protein of interest was eluted at 300 mM imidazole elution buffer. The running and elution buffers were 50 mM Tris-HCl, 200 mM NaCl buffered to pH 8.0 with the elution buffer containing an additional 500 mM imidazole. The purified protein was then desalted to 20 mM Tris-HCl, 200 mM NaCl at pH 7.4, 5% (*v*/*v*) glycerol; concentrated to approximately 10–20 mg/mL by Amicon Ultra 10-kDa centrifugal filters (Merck); flash-freeze in liquid nitrogen and stored at −80 °C until use. H111A HupZ was prepared in the same manner.

H111A mutation in HupZ was prepared using the following forward primer: 5′-GT ATT ATT GCT GTC GAG CGT ATT TTT AAT TTA C-3′. The underlined bases represent the mutational change. The reverse primer was the reverse complement of the forward primers. The insert for all constructs was verified by DNA sequencing to ensure that base changes had been introduced correctly and no random changes had occurred. All PCR products were made using QuikChange Site II Directed Mutagenesis protocol (Agilent Technologies). All necessary components were purchased from ThermoFischer Scientific.

### 4.2. Preparation of HupZ-Heme Complex

Hemin chloride (EMD Millipore, 1–5 mg) was weighed out into a Fisherbrand 1.5 mL graduated microcentrifuge tube (MCT). Then, 2.5 μL of 100% DMSO (Fisher Chemical) and 10 N NaOH (Fisher Chemical) were added to the MCT. The MCT was vortexed for 5 s before one 20 μL aliquot of 20 mM Tris-HCl, 50 mM NaCl buffered to pH 7.4 was added to the MCT. The sample was then vortexed for 10 s before the addition of another aliquot of buffer was added to the MCT. This process was repeated until 10 aliquots (200 μL) of buffer were added to the MCT. Then, 100 μL aliquots of buffer were added to the MCT and vortexed for 10 s. This process was repeated until the final volume in the MCT was 1 mL. In a 15 mL conical tube, 8 mL of buffer was added. One aliquot of 100 μL from the hemin solution in the MCT was added to the 15-mL conical tube, and the hemin solution was vortexed for 10 s. After repeating this process for ten aliquots, 1 mL of buffer was added to the MCT to remove any remaining heme adhered to the side of the MCT. Then this 1 mL was added to the 15 mL conical tube for a final volume of 10 mL. The final percentage of DMSO in buffer was 0.025%, and the final concentration of NaOH was 2.5 mM, which caused a slight increase in the pH to approximately 8.3. The hemin stock was then centrifuged before the concentration was determined using a molar extinction coefficient of ε_385_ = 58,400 M^−1^∙cm^−1^. The molar extinction coefficient of wild-type HupZ was determined to be ε_414_ = 110,000 M^−1^∙cm^−1^ and was used to determine the concentration of the heme-bound H111A variant [23].

The protein of interest was removed from the −80 °C freezer and desalted into 20 mM Tris-HCl, 50 mM NaCl buffered to pH 7.4. The absorbance was measured to determine the protein concentration, and 1.2 eq (unless otherwise stated) of hemin was slowly added to the protein. The binary complex samples were stored at 4 °C for 2 h before they were desalted into 20 mM Tris-HCl, 50 mM NaCl at pH 7.4.

### 4.3. Anaerobic Sample Preparation

Hemin was prepared anaerobically with sparged buffer (20 mM Tris-HCl, 50 mM NaCl, 5% glycerol, pH 7.4), DMSO, and NaOH. The protein of interest was purged with nitrogen and placed under an anaerobic environment. Ferrous heme-HupZ CO (Sigma-Aldrich) adducted complexes were prepared after anaerobic preparation. CO gas was placed in the headspace of the protein of interest and allowed to equilibrate for a few minutes.

### 4.4. UV-Visible Absorbance Spectroscopy

All spectra were recorded in a 1 cm, anaerobic quartz cuvette (SpectrEcology) using either a Lambda 25 spectrometer (Perkin Elmer) with a scan speed of 240 nm/s or an Agilent UV–vis. All samples were measured in 20 mM Tris-HCl buffer, pH 7.4, containing 50 mM NaCl, unless otherwise stated.

### 4.5. EPR Spectroscopy

After hemin reconstitution, the protein of interest was concentrated by ultrafiltration. The samples were transferred to quartz EPR tubes and slowly frozen in liquid nitrogen. All EPR spectra were recorded at 10 K on a Bruker E560 X-band spectrometer equipped with a cryogen-free 4 K system controlled with an ITC503S temperature controller (Oxford Instruments, Abingdon, UK) as described elsewhere [47,48,49] and an SHQE-W resonator at the 100 kHz modulation frequency, 0.6 mT modulation amplitude, and 1.002 mW power. The parallel mode EPR experiment was executed using similar conditions using a 4116 DM resonator at various modulation and microwave powers.

### 4.6. Resonance Raman Spectroscopy

After hemin reconstitution, the protein of interest was concentrated down, slowly frozen in liquid nitrogen, and shipped to Dr. Piotr Mak on dry ice for data collection. The rR spectra of ferric and ferrous CO adducts were measured using 406.7 nm and 413.1 nm excitation lines, respectively, provided by Innova 302C Kr+ laser (Coherent Inc., Santa Clara, CA, USA). The spectra were acquired using 1250M-Series II spectrometer (Horiba, Ltd., Kyoto, Japan) equipped with PyLoN:400B CCD detector (Princeton Instrument, Trenton, NJ, USA). Measurements were done using a 180° backscattering geometry, and the laser beam was focused onto the sample using a cylindrical lens. The laser power at the sample was adjusted to approximately 5 and 1–2 mW for measurements of the ferric and ferrous CO adducts, respectively. The spectral resolution was equal to 1.5 cm^−1^. The samples were contained in 5 mm OD NMR sample tubes and were spun to avoid local heating and ligand photodissociation. All measurements were conducted at room temperature. The slit width was set at 150 μm, and the 1200 g/mm grating was used. Spectra were calibrated using fenchone and acetone-d_6_ (Sigma-Aldrich, Milwaukee, WI, USA) and processed with Grams/32 AI software (Galactic Industries, Salem, NH, USA).

### 4.7. Hemin Titration

Five samples of 10 μM HupZ were mixed with 4, 8, 12, 16, or 20 μM of hemin and allowed to equilibrate overnight at 4 °C in 20 mM Tris-HCl, 50 mM NaCl buffered at pH 7.4. The UV–vis spectra were obtained via the Perkin Elmer the following day and shown in Figure S4. To account for the excess heme, the R_z_ (Soret:280, 414/280) was obtained and plotted against the amount of hemin added (inset).

### 4.8. Size-Exclusion Chromatography

All samples were reconstituted with hemin as described in the second paragraph of 4.2. All spectra were obtained on the same day on a Superdex™ Increase 200 10/300 GL in 20 mM Tris-HCl, 50 mM NaCl buffered at pH 7.4. The flow rate used was 0.4 mL/min. After column equilibration, a standard calibration curve (Figure S6) was prepared using the Gel Filtration Markers Kit for Protein from Sigma-Aldrich. After reconstitution and desalting, 200 μM of HupZ and HupZ samples (1 mL) were injected onto the SEC column.

### 4.9. Crystallization, X-Ray Diffraction Data Collection, and Refinement

HupZ and H111A variant were crystallized with conditions similar to those previously established using sitting drop vapor diffusion in crystallization plates (Hampton Research) [23]. Single crystals suitable for X-ray data collection were obtained from drops assembled with 3 μL of 5 mg/mL protein in 20 mM Tris-HCl pH 7.4 buffer containing 50 mM NaCl layered with 3 μL reservoir solution containing 0.2 M lithium acetate, 20% PEG 3350. The trays were sealed and moved to a vibration-free, 22 °C crystallization incubator (Molecular Dimensions, Altamonte Springs, FL, USA). Crystals appeared within 48 h and were cryo-cooled with a cryo-protectant containing 30% glycerol in addition to the mother liquor. The H111A variant was crystallized in the same manner. The only difference was the mother liquor, which was 0.1 M citric acid, 1.3 M ammonium sulfate at pH 3.4. X-ray diffraction data were collected at the beamline station 9–2 of SSRL. All data collections were performed at 100 K. The diffraction data were indexed, integrated, and scaled with HKL-2000 [50].

### 4.10. Heme Degradation Activity Assay

All activity assays were performed similarly to previously described [23] with a few minor modifications. After preparation of the binary complex, HupZ-heme (10 μM) was mixed with 200 μM of NADPH, 0.4 μM of CPR, and 1 μM of catalase. Each absorption spectra were obtained at 10 min intervals on the Agilent UV–vis spectrophotometer in 20 mM Tris-HCl pH 7.4 buffer containing 50 mM NaCl. The heme degradation activity assay for H111A HupZ utilized 100 μM of NADPH. All spectra were obtained with the Perkin Elmer UV–vis spectrophotometer.

## 5. Conclusions

In summary, a comprehensive biochemical, spectroscopic, and structural characterization led us to propose that the observed heme binding in HupZ is through its His_6_-tag in a sandwiched di-heme complex from two subunits with an oxygen-derived bridging ligand (Scheme 2). This work serves as a caution against tags, in particular the widely used His_6_-tags, when characterizing heme-binding proteins. The heme ligation to the *C*-terminal His_6_-tag is noticed to resemble a regional structure similar to that of genuine heme oxygenase proteins, thus leading to a minor heme degradation activity. It should be noted, however, that the side product is CO, rather than the expected formaldehyde. Therefore, the observed weak heme-degradation activity is an added function. Should HupZ be a new member of HO in the IsdG family, formaldehyde is the expected product. A continued study on a non-tagged HupZ protein is needed to define its endogenous biological function in the heme utilization pathway of GAS.

## Figures and Tables

**Table 1:** UV–vis characteristics of heme-HupZ-V5-His_6_ complex in different oxidation states and H111A variant.

Identity	Soret Band (nm)	α Band (nm)	β Band (nm)	Charge Transfer (nm)
Free ferric heme (hemin)	385	548	515	649
Ferric heme-HupZ complex	414	564	536	-
Ferrous heme-HupZ complex	424	559	530	-
Ferrous CO-heme-HupZ complex	421	567	537	623
Ferric CN-heme-HupZ complex	422	543	-	-
Ferric heme-HupZ H111A complex	414	568	531	-

**Table 2:** X-ray diffraction data collection and refinement statistics.

	HupZ-V5-His_6_	H111A HupZ-V5-His_6_
**Data Collection**		
Wavelength (Å)	0.97913	0.97946
Space group	*P*2_1_2_1_2_1_	*P*6_5_22
Cell dimensions		
a, b, c (Å)	40.0, 62.4, 94.9	54.9, 54.9, 333.3
α, β, γ (°)	90, 90, 90	90, 90, 120
Resolution (Å)	50.00–1.70	50–1.97
	(1.73–1.70) ^a^	(2.00–1.97)
Total reflection	258352	661695
Unique reflection	26686	22490
Redundancy	9.7 (9.9)	29.4 (32.0)
*R*_sym_ or *R*_merge_^b^ (%)	5.9 (18.3)	17.1 (97.2)
CC_1/2_^e^	99.8(98.6)	98.8 (90.6)
I/σI	40.9 (9.7)	25.4 (3.6)
Completeness (%)	99.7 (99.9)	99.9 (100)
**Refinement**		
Resolution (Å)	37.8–1.70	38.71–1.98
No. reflections	26636	21371
*R*_work_^c^/*R*_free_^d^ (%)	18.36/21.87	19.54/22.65
No. Atoms/*B*-factors (Å^2^)		
Protein (Chain A)	985/27.0	945/34.4
Protein (Chain B)	994/27.9	959/34.3
Water (Chain S)	208/34.7	161/40.9
r.m.s. deviations ^e^		
Bond lengths (Å)	0.007	0.009
Bond angles (°)	1.056	1.012
Ramachandran Statistics ^f^		
Favored (%)	96.8	98.3
Allowed (%)	3.2	1.7
Outlier (%)	0.00	0.00
**PDB Code**	**7KPZ**	**7KQ2**

^a^ Numbers in parentheses refer to data in the highest resolution shell. ^b^ *R*_merge_ = Σ|I_h_ − <I_h_>|/Σ I_h_, where I_h_ is the observed intensity and <I_h_> is the average intensity. ^c^ *R*_work_ = Σ ||*F*_obs_| − k|*F*_cal_||/Σ|*F*_obs_|. ^d^ *R*_free_ is the same as *R*_obs_ for a selected subset (10%) of the reflections that were not included in prior refinement calculations. ^e^ According to Engh and Huber [27]. ^f^ Calculated by using MolProbity [28].

## Data Availability

The X-ray structures were deposited in the Protein Data Bank under accession codes 7KPZ and 7KQ2.

## Supplementary Materials

Table S1: SEC peaks of wild-type HupZ and H111A variant with and without heme bound, Figure S1: Low-temperature (10 K) parallel mode EPR analysis of wild-type HupZ-heme, Figure S2: HupZ-heme complex (black) titrated with NaCN, Figure S3: Aerobic and anaerobic reconstitution of HupZ with hemin, Figure S4: Heme titration of HupZ, Figure S5: EPR and UV–vis spectroscopic analysis of H111A variant, Figure S6: Activity assay of wild-type HupZ-heme complex and Figure S7: Calibration curve of standard proteins in MWGF200 Kit on Superdex 200.

## Sample Availability

Not available.
