# Supplementary material for: Heme Binding to HupZ with a C-Terminal Tag from Group A Streptococcus
Source: Molecules. 2021 Jan 21;26(3):549. doi: 10.3390/molecules26030549 (PMC7865249; doi:10.3390/molecules26030549)
Supplement: Supplementary file 1 [file molecules-26-00549-s001.pdf]

Heme Binding to HupZ with a C-Terminal Tag from Group A Streptococcus

Ephrahime S. Traore,[†] Jiasong Li,[†] Tapiwa Chiura,[‡] Jiafeng Geng,[¶] Ankita J. Sachla,[‖] Francis Yoshimoto,[†] Zehava Eichenbaum,[‖] Ian Davis,[†,¶] Piotr J. Mak,[‡,*] and Aimin Liu,[†,¶,*]

† Department of Chemistry, The University of Texas at San Antonio, San Antonio, TX 78249, United States
‡ Department of Chemistry, Saint Louis University, St. Louis, MO 63103, United States
¶ Department of Chemistry, Georgia State University, Atlanta, GA 30302, United States
‖ Department of Biology, Georgia State University, Atlanta, GA 30302, United States
* Correspondence: piotr.mak@slu.edu or Feradical@utsa.edu

Table S1. SEC peaks of wtHupZ and H111A variant with and without heme bound.

Protein	Peak 1 (mL)	Oligomeric State	Peak 2 (mL)	Oligomeric State	Peak 3 (mL)	Oligomeric State
HupZ	-	-	-	-	18.0	Dimer
HupZ-heme	-	-	14.3	Heptamer	18.6	Dimer
H111A	-	-	-	-	17.7	Dimer
H111A-heme	10.0	N/A*	15.1	Pentamer	18.6	Dimer

*The molecular weight did not fall in the range of the standards used; thus, it could not accurately be determined.

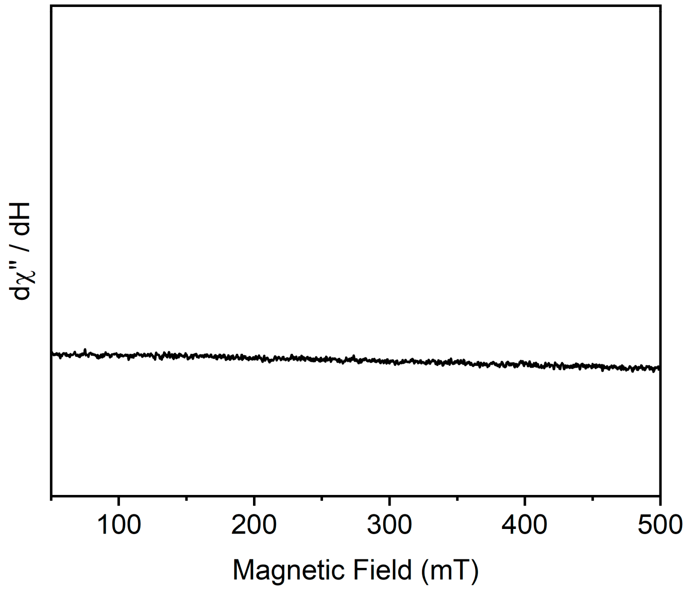

Figure S1. Low-temperature (10 K) parallel mode EPR analysis of wild-type HupZ-heme (protein concentration 250 μM) using a 4116 DM resonator as described in the Materials and Methods.

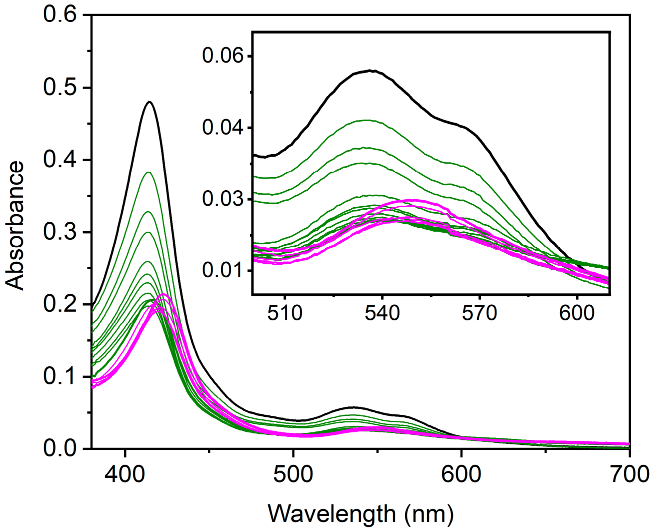

Figure S2. 5 µM HupZ-heme complex (black) titrated with NaCN to 2 mM (thick green) and 80 mM (thick pink), respectively, with the intermediates shown as thin green and thin pink lines. The green lines correspond to the first phase of CN binding, and the pink lines represent the second phase.

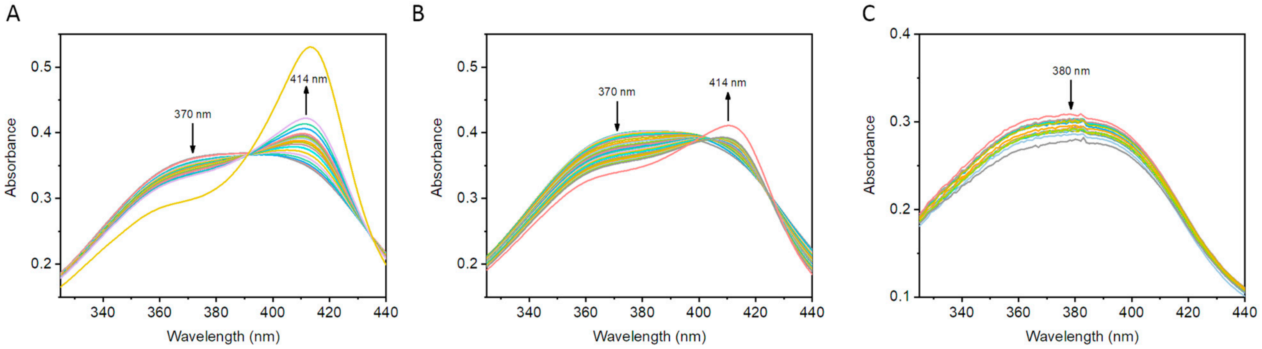

Figure S3. Aerobic and anaerobic reconstitution of HupZ with hemin. (A) Aerobic, (B) anaerobic, and (C) strictly anaerobic reconstitution. All spectra were prepared by mixing 5 µM of HupZ with 5 µM of hemin. Each spectrum was obtained at 5-minute intervals.

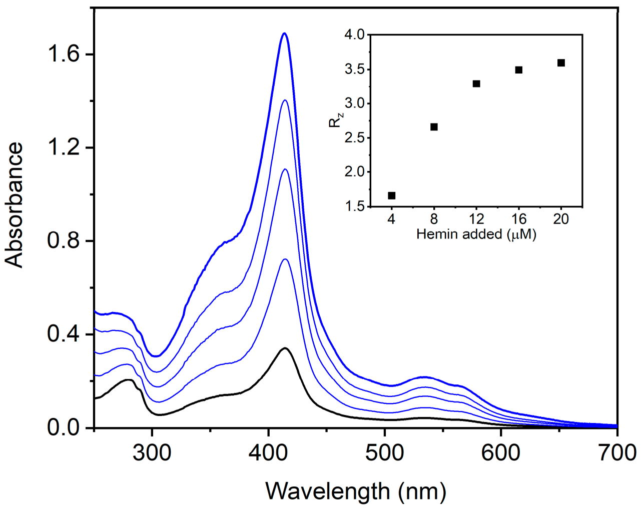

Figure S4. Heme titration of HupZ. (A) UV-vis spectra of 10 μM HupZ titrated with 4, 8, 12, 16, and 20 μM of hemin from black to blue. The inset shows the 414/280 ratio of each titration graphed against the concentration of heme added. Once 10 μM, or 1 eq of heme was added, the R_z value began to plateau, suggesting that HupZ binds at a 1:1 ratio of protein:heme.

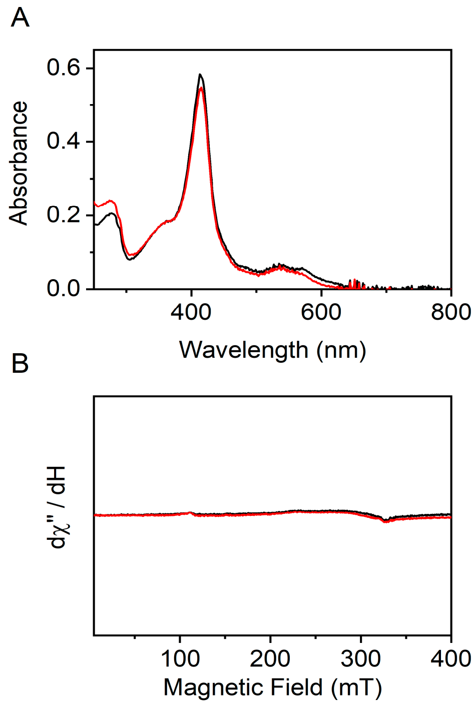

Figure S5. Spectroscopic analysis of H111A variant. UV-vis spectra (A) and EPR spectra (B) of wild-type HupZ (black) and H111A HupZ variant (red). The concentration used for both UV-vis spectra were 5 μM for both wt and H111A HupZ. The concentration for both EPR spectra in this set of experiments was 200 μM.

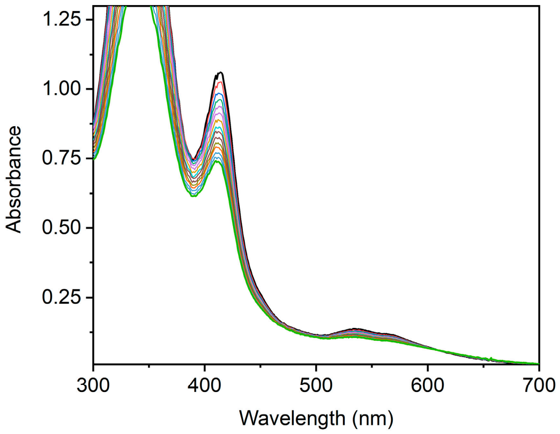

Figure S6. Activity assay of wild-type HupZ-heme complex. 10 μM of HupZ-heme was mixed with 200 μM of NADPH, and 0.4 μM of CPR. Each spectrum was obtained at 10 min intervals for a total of 2 h.

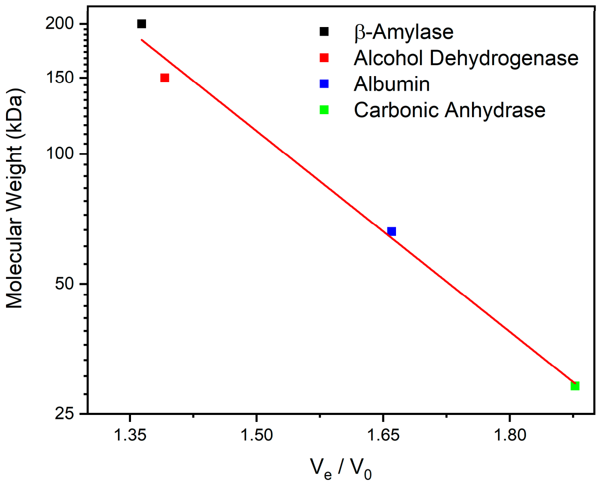

Figure S7. Calibration curve of standard proteins in MWGF200 Kit on Superdex 200. The standard proteins in the plot are β-amylase (200 kDa), alcohol dehydrogenase (150 kDa), albumin (66 kDa), and carbonic anhydrase (29 kDa), respectively. The resulting linear line has a slope of 1.5505 and a Y-int of 4.37913.
